# A Novel Social Distancing Approach for Limiting the Number of Vehicles in Smart Buildings Using LiFi Hybrid-Network

**DOI:** 10.3390/ijerph20043438

**Published:** 2023-02-15

**Authors:** Sallar Salam Murad, Salman Yussof, Rozin Badeel, Wahidah Hashim

**Affiliations:** 1 Institute of Informatics and Computing in Energy, University Tenaga Nasional, Kajang 43000, Malaysia; 2 Department of Network, Parallel & Distributed Computing, University Putra Malaysia, Seri Kembangan 43400, Malaysia

**Keywords:** LiFi, energy, pandemic, social distancing, smart building, vehicle scheduling, V2I

## Abstract

The coronavirus (COVID-19) has arisen as one of the most severe problems due to its ongoing mutations as well as the absence of a suitable cure for this virus. The virus primarily spreads and replicates itself throughout huge groups of individuals through daily touch, which regretfully can happen in several unanticipated way. As a result, the sole viable attempts to constrain the spread of this new virus are to preserve social distance, perform contact tracing, utilize suitable safety gear, and enforce quarantine measures. In order to control the virus’s proliferation, scientists and officials are considering using several social distancing models to detect possible diseased individuals as well as extremely risky areas to sustain separation and lockdown procedures. However, models and systems in the existing studies heavily depend on the human factor only and reveal serious privacy vulnerabilities. In addition, no social distancing model/technique was found for monitoring, tracking, and scheduling vehicles for smart buildings as a social distancing approach so far. In this study, a new system design that performs real-time monitoring, tracking, and scheduling of vehicles for smart buildings is proposed for the first time named the social distancing approach for limiting the number of vehicles (SDA-LNV). The proposed model employs LiFi technology as a wireless transmission medium for the first time in the social distance (SD) approach. The proposed work is considered as Vehicle-to-infrastructure (V2I) communication. It might aid authorities in counting the volume of likely affected people. In addition, the proposed system design is expected to help reduce the infection rate inside buildings in areas where traditional social distancing techniques are not used or applicable.

## 1. Introduction

### 1.1. Background

Due to the coronavirus disease of 2019 COVID-19, international attitudes toward the pandemic disease have shifted, with major ramifications for worldwide health and the economy [1,2]. According to the World Health Organization (WHO), more than ten million individuals have been verified to be contaminated in 210 nations, with over 500,000 deaths [3]. COVID-19 has resulted in massive economic damage in addition to the international health crisis (e.g., a projected 25% jobless rate in the United States) [4]. The pandemic has affected network connections and caused data rate fluctuations around the world, including in Malaysia [5]. Stopping the transmission of the disease, reducing its bad effects, and buying further opportunities for the creation of pharmacological remedies are all urgently required in such a scenario. The term “social distance of social distancing (SD)” refers to strategies that seek to mitigate disease spread by reducing the frequency and severity of human physical encounters, preventing big gatherings, and preserving a safe distance among individuals [6]. SD can be an effective non-pharmaceutical strategy to restrict the spread of dangerous diseases such as severe acute respiratory syndrome (SARS), Influenza A virus subtype (H1N1), and COVID-19 [7,8,9].

SD slows the growth and consequences of the disease since it diminishes the probability of an infected individual transmitting the infection to a healthy person. SD is critical in the initial stages of a pandemic to halt the spread of the disease. Different strategies can play a major role in lowering the infection rate and postponing the development of the disease. As a result, the burden on healthcare systems is decreased, as are death rates. The sooner SD is implemented, the more intense the effects would get [10]. To combat the spread of the recent COVID-19 pandemic, many nations have implemented SD measures, such as travel restrictions, the closure of public venues, and cautions advising members of the public they must retain a 1.5–2 m distance while walking outside [11,12,13]. Such massive initiatives, nevertheless, can be tough to accomplish; for instance, citizens still need to walk outdoors to purchase food, access health care, or perform essential employment since not all public areas can be sealed.

In this regard, technology plays a significant role in assisting SD practices. It works, for example, by detecting the distances among individuals and sounding an alarm when they are too near to each other. There are two broad classes of SD techniques: public and private. For the public population, this entails blocking or limiting entry to professional institutions and workplaces, prohibiting mass meetings, and establishing travel restrictions, in addition to quarantine and border control centers. Private measures involve separation and isolation, besides advising people to keep physical limits among them [6]. SD, despite its enormous potential, is ultimately successful whenever deployed correctly. Nevertheless, it is not a simple process to achieve due to multiple difficulties, along with the following [14]: (i) certain SD scenarios could produce a negative influence on the economy, (ii) specific SD systems violate personal rights, such as data privacy, people’s locations, and privacy, (iii) people’s behaviour is hard to change, and (iv) difficulties arise whenever there is a high population density. The approach presented in this article for the SD problem seeks to minimize these difficulties.

**Figure 1:**
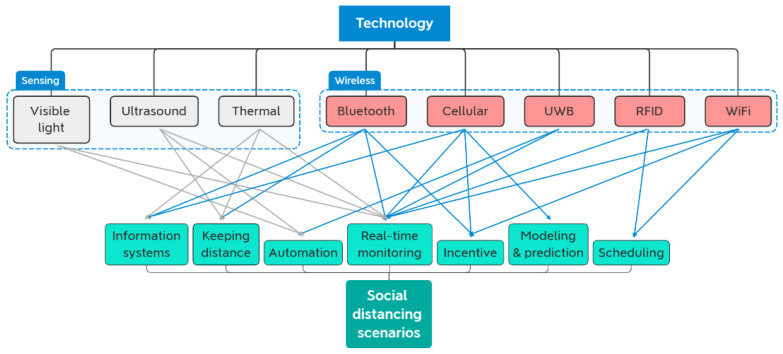
Recent wireless and emerging technologies used for different social distancing scenarios.

Social separation may not be as simple as physical separating due to the complexity of viruses and the rapid rise of social contact and globalization. It includes a wide range of non-pharmaceutical actions and initiatives, such as surveillance, diagnosis, and public warning, that aim to limit the spread of infectious illnesses. In the adapted settings, keeping a safe distance between people (e.g., 1.5 m) can be facilitated by a variety of solutions. Many mobile and related solutions can be employed for real-time monitoring of crowds and public venues. In order to keep everyone in public circumstances at a harmless distance (e.g., 1.5 m), various wireless technologies have been adopted. According to [14], wireless and developing technologies monitor individuals and public places in real-time, helping to keep distance, schedule traffic into buildings, and perform contact tracing, crowd detection, prediction, and other scenarios, and have been employed in various situations for different purposes; for example, some systems trigger an alarm (keeping distance), limit access (scheduling/monitoring), and others, as shown in Figure 1.

Such surveillance is needed to collect useful data (e.g., records of building occupants, interactions, indications, gatherings, and violations of social distancing rules) in order to ease social distance. Technologies like bluetooth (BLE), ultra-wideband (UWB), and thermal sensors provide the means to monitor the spread of infection and locate sick individuals, as well as the contacts that they established. If they have been exposed to infectious people, they can take precautions (e.g., self-separation, limiting contact, and testing for the disease). Wireless fidelity (WiFi) and radio frequency identification (RFID) are two other technologies that can be used to control when people enter and leave a place. Using cutting-edge tools, we can gather information about pandemics. Based on the data, we can determine who will become infected and who will be susceptible. The use of SD scenarios varies widely. Up until this point, real-time monitoring and maintaining a safe distance have been the most common case studies, while scheduling and incentives have been employed less frequently [14].

### 1.2. Problem Formulation

The fundamental replica ratio (*Ro*), which indicates on a median how many individuals a case (i.e., an infectious person) would infect during its entire contagious time, is one of the major parameters for SD measures selection [15]. The new SD system design presented in this study aims to reduce the *Ro*. For instance, *Ro* < 1 means that each case may infect less than one person, suggesting that the disease is dropping in the group under consideration. Because the amount of *Ro* reflects the rate at which the disease advances, it has been one of the key essential indications for SD measure choosing [8,9]. Mathematically speaking, *Ro* can be determined by:

(1) $$Ro=\int_{0}^{\infty }b\left(a\right)F\left(a\right)da,\;$$

where *b*(*a*) is the median volume of fresh cases infected by an infected person per unit of time throughout the infectious time *a*, and *F*(*a*) is the likelihood that the person will remain contagious throughout the period *a* [15]. A typical manner for evaluating the productivity of SD is to portion the outbreak ratio, which is the proportion of infected individuals in an exposed group. Diverse kinds of social distancing techniques may have different degrees of efficiency in preventing the disease from spreading.

Various approaches have been shown to be beneficial in decreasing infection rates including (i) facility closing procedures, (ii) isolation of proven cases as well as cases demonstrating related symptoms, and (iii) household quarantines. Studies have revealed that if the level of adherence is high enough, such practices can be effective. Controlling the number of individuals exclusive to a building, such as a shop, shopping mall, or university, is the best way to use light fidelity (LiFi) technology in SD. Unfortunately, current SD methods make it nearly impossible to recognize and monitor individuals inside automobiles before entering buildings. For this study, with various access points (APs) implemented at the building’s gates, the number of vehicles currently inside the building can be estimated based on the data obtained at the entry permit requests that come from user devices (vehicles) towards the APs (at the gates).

### 1.3. Motivation and Contribution

Citizens and governments find it challenging to deal with SD. There are numerous obstacles, such as establishing and maintaining a suitable distance of 1.5–2 m among individuals on all occasions, especially in the healthcare, government, and delivery services sectors. In addition, working from home [16] is not always possible since essential services must continue to run even if a pandemic occurs. Furthermore, even when SD is in operation, automobiles such as cars and motorcycles are permitted to travel in certain private places such as:

I. Companies: especially those who provide shipping services;
II. Hospitals: including ambulance and emergencies;
III. Healthcare facilities: including drive-through services and medical suppliers;
IV. Government: including police and military.

Therefore, our system design proposed in this research is designed to support these private places when a pandemic occurs.

In our recent study [14], a review of the most recent studies of various systems and models using different wireless and emerging technologies for SD in the time of COVID-19 was presented. In summary, SD scenarios consider human factors only for measurements and precautions in indoor and outdoor environments and settings. To the best of our knowledge, no SD model/system has been found so far that considers the SD of vehicles such as cars and motorbikes inside smart buildings.

To address the aforementioned challenges, this study proposes a new SD solution and a new perspective on using LiFi technology [17]. Since human engagements are vastly greater indoors than outdoors, the suggested design in this study is built and designed for smart buildings that promote technology adoption. This study focuses on using LiFi technology in a new SD system design for vehicle access scheduling and real-time monitoring scenarios. LiFi is being used for an SD system for the first time in this study. Moreover, the specifications and features of LiFi technology have been proven to outperform other technologies in terms of speed, data transmission, security, and interference [18], which was the reason behind choosing it over other radio frequency (RF)-based wireless technologies in this study. Using LiFi technology over others such as BLE, WiFi, or RFID has several benefits (shown in Table 1).

From the unique characteristics of LiFi, we can observe that some features such as speed are essential for the proposed design since the movement and entry of vehicles require quick response and transmission of data. However, the amount of the exchanged data is not large, which means that using an alternative technology might lead to the latency of transmission. For example, using BLE at the entry of vehicles would take some time for pairing the devices. Moreover, it should be noted that LiFi is not cheap and not popular at this time. However, a few companies have developed a few types of LiFi modules and it is expected to be available and popular in the coming years. The lack of interference with other wireless technologies makes the use of LiFi in the proposed social distancing system design for deployment possible in various places, especially where RF technologies are forbidden to be used. In addition, the existing infrastructure of lightning makes it easier for setting up the LiFi APs. Since all vehicles are registered and recognized by the organizations that would adopt the proposed system, all drivers of permitted vehicles are responsible for using the system while taking care of some restrictions. For example, when taking the pandemic situation and infection precautions into account, drivers should not allow unregistered/unauthorized individuals using the permitted vehicles to access the building through their validity of using the system since only drivers of vehicles are assigned to their vehicles as per the safety regulations.

The proposed system design is considered a real-time procedure, wherein the interaction of system components would prevent it from being vulnerable to being manipulated by unauthorized behaviour. For example, using a phone-based application with a BLE application instead of LiFi APs for data transmission and authentication could result in granting vehicles access to the building by a third-party user. This study aims to provide an effective system design that is mainly focused on monitoring and detecting people while driving their vehicles as well as performing access scheduling and tracking for vehicles in order to maintain distance and reduce the density of vehicles and people inside the building as explained in the next section.

The rest of this paper is organized as follows: Section 2 shows the literature review. Section 3 presents the methodology including the system model and the proposed algorithm. The results and discussion are presented in Section 4. Section 5 discusses some challenges and limitations. Finally, the conclusion of this study is shown in Section 6.

## 2. Literature Review

Because of the exponential development of emerging technologies, a number of innovative approaches are available, which can provide conducive conditions for social alienation. Similar works that explored the use of wireless technologies for psychological distance are discussed here. Distancing strategies were demonstrated to reduce the daily reports of incidents. Social isolation can reduce the number of incidents and save public health expenditures [8]. In addition, social isolation can help lower the number of affected people in the long run.

Several governments have enacted social distancing measures, including travel restrictions, putting an end to public gatherings, and instructing people to maintain a 1.5–2 m spacing while outside [11,12,13]. In this scenario, technology aids social alienation. It senses people’s distances and alerts them when they go too close. As shown in [19,20], cellular, global navigation satellite system (GNSS), artificial intelligence (AI), and Thermal can be utilized in many contexts, however, Zigbee, RFID, visible light (VL), and Ultrasound are not. In this section, two groups of studies will be presented. The first group will discuss vehicle-free studies, while the other group will include studies that considered the use of vehicles and/or buildings in their scenario or model.

### 2.1. Vehicle-Free Social Distancing-Related Studies

Various SD scenarios were used, according to [14]. The most common use cases so far have involved real-time monitoring and maintaining a safe distance, whereas scheduling and incentive have been employed much less frequently. Specifically, in the research [21], MQTT was used to detect temperature and saturation status. This research uses MQTT to categorize individuals by temperature and saturation.

Body-induced thermal signatures for proximity and temperature detection were studied [22]. A statistical approach captures body-induced thermal signatures from noisy data, while a mobility model detects multi-body activities and minimizes false target detection. Based on a mathematical model and Bayesian decision concept, the suggested screening approach allows the user to wander during the screening operation while its location is calculated (constantly). This method can be used in the context of multi-sensory installations and large infrared radiation (IR) arrays.

The study in [23] describes a multi-sensor Internet of Things (IoT) system for smart city social distancing. This study aimed to provide an IoT system based on an IoT, wireless sensor network (WSN), and the techniques neural network (NN) and shortest path first (SPF)) that could indeed distinguish cautions, obtainable exits, gathering locations, fairest and shortest pathways, and overcrowding based on real-time data gathered by sensors and cameras using the IoT system for emergency and monitoring.

The authors of [24] used AI, a mass surveillance platform, and a beyond fifth-generation (B5G) architecture utilizing images from chest X-rays or CT scans to diagnose COVID-19 and developing a nationwide surveillance network to track people’s attempts to avoid contact with others, the prevalence of mask use, and core body temperature.

Technology-assisted boarding distance calculation was explored in [25]. It highlighted COVID-19’s distance estimate vulnerability, which might lead to errors in distance computation and the impracticality of standard monitoring approaches throughout passenger boarding/deboarding. The study in [26] suggested a distance violation model. The research developed an IoT edge-based approach for sensing breaks in indoor social distance requirements. The system design captures group members’ status based on specified distances.

The study in [27] examined mass gatherings across boundaries. This sort of connected approach can identify global infectious disease concerns, offer information on which illnesses are likely to spread to the mass gathering (MG), and provide anticipatory surveillance at the MG. It permits mathematical modelling of serious illness spread to and from MGs, simulates community health metrics at regional and worldwide levels, and facilitates interaction between the scientific sector and stakeholders on a national, global, and worldwide level.

AI was used for prediction in [28]. It monitors users’ health data in real-time to anticipate COVID-19 infection by observing indicators, then sends them urgent warnings, medical evaluations, and important warnings. It uses patient IoT gadgets to capture critical hospital/quarantine data. It cautions officials to limit chronic illness spread and respond quickly.

The distance studies contain comparable components but different settings. The study in [29] introduced Suraksha, an intelligent wearable device that helps SD while walking outdoors. It is a simple electronic device that is easy to use. The gadget can track contacts and wellness applications via BLE. The research [30] devised a mechanism for detecting SD at university campuses using microcomputer modules and mobile hubs as entry permits. A monitoring center receives mobile node data.

In [31], the “6Fit-A-Part” protocol was presented as a means of achieving real-world separation between two wearable devices. The research focused on developing a wearable device that sounds an alarm when it detects another sensor of the same sort within a given range. Utilizing commercially available UWB wireless technology, it can make accurate, real-time distance estimates relative to other nearby users.

The study in [32] examined the viability of employing indoor localization to assess user distances. A standard indoor localization system (ILS) design with three use-cases was proposed. The authors provided a framework for measuring the suggested architecture’s functionality.

### 2.2. Social Distancing-Related Studies with Vehicles or Buildings

The COVID-19 pandemic outbreak has made it necessary for everyone to practice social distancing, wear masks, and perform routine hand sanitization. Currently, hospitals must be sanitized by a human workforce, which is one of the more difficult tasks because of the high risk of infection. Therefore, it is crucial to minimize human involvement and instead use robotic, vehicular, smart building, or smart city systems for these prerequisites.

The authors of [33] present a prototype of an Arduino-powered hospital sanitization robot vehicle (HSRV) that can be remotely controlled by hospital staff to clean any area of the facility without endangering their safety. The robotic concept can be utilized in a variety of settings, including hospitals, homes, COVID care centers, and quarantine centers, to monitor patients who are either symptomatic or asymptomatic while they are isolated.

The robot operating system (ROS) mimics a retail mall using Gazebo in the research [34], and Turtlebot and unmanned aerial vehicles (UAV) keep an eye on the shoppers. A specific individual is recognized and followed around the shopping mall by Turtlebot, which does this by analyzing the frames it has captured. The wheeled robot known as Turtlebot follows people around without making physical contact and can be used as a shopping cart. As a result, a customer does not come into contact with the shopping cart that someone else has used, which not only makes shopping easier for that customer but also protects them from potential contamination. UAVs can spot people from above and calculate the distance between groups of people as it does so. In this manner, a warning system can be developed by locating the various locations where social distance is ignored. Turtlebot makes use of a histogram of oriented-gradients (HOG)-support vector machine (SVM) in order to identify people, and Kalman Filter is applied in order to follow people. The purpose of this paper is not to aid in the early diagnosis of infection, but rather to aid in the suppression of the spread of COVID-19 in public settings. Some of the research has focused on the effectiveness of social distance COVID-19 mitigation.

Drones, or UAVs, will have a profound impact on society and the way we live. To increase the use of UAVs in commercial and civil applications, new ideas and effective deployment are needed. There is still a lack of a roadmap allowing network service providers to meet the target service level agreement requirements, which presents a number of design challenges for UAV-assisted applications.

The article [35] proposed a comprehensive framework for the UAV as a service paradigm, bringing together all the actors/stakeholders involved in providing the UAV-augmented service and depicting their communication through data/service/money streams. The authors then apply their framework to the pandemics caused by viruses like SARS-CoV-2 and discuss how it can be used to enforce social isolation, spray disinfectants, broadcast warnings, transport medicine, and improve monitoring efforts.

One of the most crucial functions of the cities of the future will be the management of citizen and community safety. Because huge crowds might enhance the danger of illness spread, crowd observation and surveillance is extremely vital in the present COVID-19 pandemic situation. However, there are a number of difficult technical challenges associated with crowd tracking, such as precise headcounts and protecting individuals’ anonymity.

In [36], a new intelligent method is proposed for monitoring outdoor crowd density using a tile-map-based approach that is consolidated with the cloud of things and data analytics. The suggested technology analyses crowd activity to create contingency strategies, decrease the number of accidents, increase public safety, and create a smart city. UAV-mounted sensors were used for both the density calculations and the final visualization.

**Figure 2:**
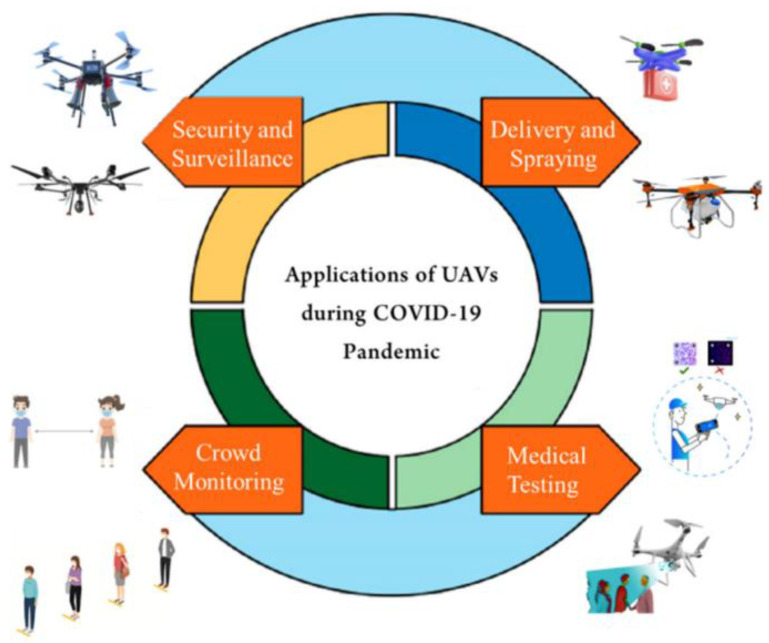
Role of UAVs in COVID-19 pandemic [37].

The research in [37] investigates several potential technologies that could be used to lessen the risk posed by COVID-19. This includes UAVs. The potential for UAVs to transport medical supplies, spray disinfectants, broadcast communications, conduct surveillance, inspect facilities, and screen patients for infection are intriguing. This article takes a close look at the pros and cons of using drones for medical purposes. UAVs are unmanned aircraft that can be remotely piloted. Microprocessors, sensors, and other devices allow UAVs to operate independently and be controlled from a distance. Connectivity options allow UAVs to link up with satellites or remote ground control stations (GCSs) such as smartphones and computers. A human operator must use a remote control to guide the UAV’s movements remotely. UAVs are strongly encouraged wherever the availability or safety of human intervention is severely constrained. Different types and sizes of UAVs have been designed for specific jobs. Different UAV monitoring methods may not be able to perform each of these tasks as efficiently as others. Presently, UAVs are used for a wide variety of purposes (illustrated in Figure 2) in the fight against COVID-19, including the delivery of vaccines and medical kits, the dissemination of public announcements, the monitoring of large crowds, the spraying of disinfection, the screening of large numbers of people, the monitoring of the crowd from above, and the transportation of vaccines and other clinical supplies.

In order to slow and eventually stop the spread of the coronavirus during the COVID-19 pandemic, monitoring social distancing in public spaces is essential. In order to detect unsafe social distance between people, the system presented in [38] uses a camera-equipped drone to apply deep learning algorithms, specifically the YoloV4 CNN algorithm, to detect persons in images, combine this with transformation equations to calculate the real-world position of each person, and finally calculate the distance between each pair to determine whether it is safe.

Drones can identify a group of people who are unmasked and not keeping their distance from one another. For the purposes of mask detection and social distance monitoring, a drone equipped with deep learning was developed in [39]. The primary function of this Raspberry Pi 4-based system is to monitor devices connected to the IoT in industrial settings. This automated drone system uses speaker alerts to keep a safe distance from the crowd. Using a faster region with a convolutional neural network (faster R-CNN) model, this system can take pictures and identify unmasked people in them. When the system identifies an individual without a mask, it notifies the appropriate authorities and the nearest police station. The developed model is comprehensive in that it can detect faces across a wide range of benchmark data.

### 2.3. Discussion

**Figure 3:**
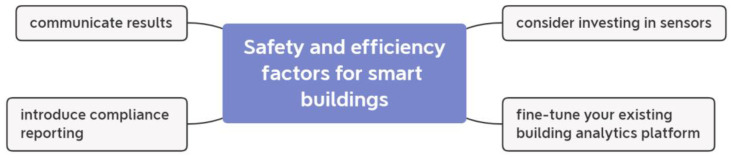
Important factors for keeping safety and efficiency during pandemics.

During and after the COVID-19 epidemic, building analytics will be an invaluable tool for ensuring the smooth and secure operation of any facility. Data from the building analysis system (BAS) is used in building analytics. As a result, smart analytics solutions will be able to efficiently collect this data from the buildings and process millions of data samples to deliver responses. Maintaining building functionality during and after the COVID-19 epidemic is essential; the authors suggested four measures in [40]. The factors in question are depicted in Figure 3.

As such, it is crucial that the building analytics platforms are properly configured so that they can be used for compliance reporting in addition to arc-fault detection devices (AFDD) and energy reporting. Buildings that are open with their performance data and maintain open lines of communication with their various stakeholders, including the building’s occupants, are more likely to stand out.

Given the severity of the current pandemic, it is imperative that hospitals and other medical facilities be made as sanitary as possible. Research into the building automation and control system (BACS) at three Danish healthcare facilities was conducted in [41]. Because of the unique pressures placed on healthcare facilities during a public health emergency like the COVID-19 outbreak, this case study was selected. Such buildings cannot function without building automation and control systems that have been meticulously planned, installed, and maintained. The purpose of the research was to have a conversation about how the building automation upgrade affects energy efficiency. After gathering the existing BACS auditing data for three representative buildings, the study’s authors simulated three potential enhancements to the simulated buildings’ automation systems: (1) technical enhancements, (2) occupant comfort enhancements, and (3) energy efficiency enhancements. Energy poverty has been made worse by the pandemic due to the double whammy of lockdowns, which have increased energy costs while simultaneously reducing economic activity and lowering living standards. Alterations in the natural environment, however, may present promising openings for the development of locally generated renewable energy sources, energy storage systems, and transmission infrastructure.

Some of the above-mentioned technical solutions, such as robots for cleaning, surface disinfection, temperature measurement, and sensors for infection demonstrating, have been established in direct response to the COVID-19 plague. The potential for smart buildings to offer solutions to several problems brought on by the COVID-19 pandemic has been discussed at length above. Duality exists in all things, including smart structures. If smart buildings are to effectively enhance the comfort and security of their occupants, they will need to gather extensive information about their habits, including potentially sensitive information. As a result, one of the most pressing challenges facing the management and operation of smart buildings is the need to ensure the privacy of occupants’ data.

By 2020, the world’s smart buildings had amassed over 37 zettabytes of data from an array of sensors and Internet-enabled gadgets [42]. Concerns about data breaches and other security issues can be allayed by taking privacy and security measures during the initial setup of a smart building system in accordance with the general data protection regulation (GDPR) and any applicable national data protection legislation. In addition, work must be done to reduce the amount of data needed to collect in order to enhance the analysis method/algorithm. After all, as data volumes increase, it becomes more challenging to ensure proper procedures have been followed when processing the data.

The question of who should foot the bill for upgrading to smart technologies in existing buildings is another major concern with the smart building movement. The quality of life in such a building is enhanced by its increased technological sophistication, but this comes at a higher price. This begs the question of whose responsibility it is to foot the bill for the upcharge. Although landlords may shoulder the upfront cost of smart technology, they may later pass it on to tenants and/or occupants in the form of rent increases that may be unpopular. To resolve this discrepancy, consider that using smart technologies can cut down on energy use and utility costs while also helping to stop the spread of infectious diseases like COVID-19. This consideration is growing in significance as we learn more about the impact of buildings on people’s health. Though this will not stop the spread of COVID-19 or any other infectious disease right away, it is a long-term solution that could improve public health and even strengthen the immune system of the population.

Many people are concerned that because smart buildings are still a novel concept, there are not yet many regulations and laws in place to restrict the activities associated with them. A more complete and reasonable set of regulations and levels of supervision will emerge over time, despite the fact that the concept of the smart building is novel. The benefits of smart building technology continue to outweigh the drawbacks. Global real estate firm BentallGreenOak (BGO), the united nations environment programme finance Initiative (UNEP FI), and the non-profit center for active design (CfAD) collaborated on a study titled “A New Investor Consensus: The Rising Demand for Healthy Buildings,” which found a growing business case for healthy buildings around the world. To be more specific, certified healthy buildings saw rent increases of 4.4% to 7.0% compared to their non-certified equivalents.

Businesses located in such structures enjoy greater job satisfaction, increased productivity, decreased absenteeism, and retention of valuable employees [43]. Smart building is an emerging economic sector with promising prospects even in the aftermath of a global pandemic. In Table 2, we have compiled a synopsis of the most important articles in the relevant literature.

## 3. Methodology

**Figure 4:**
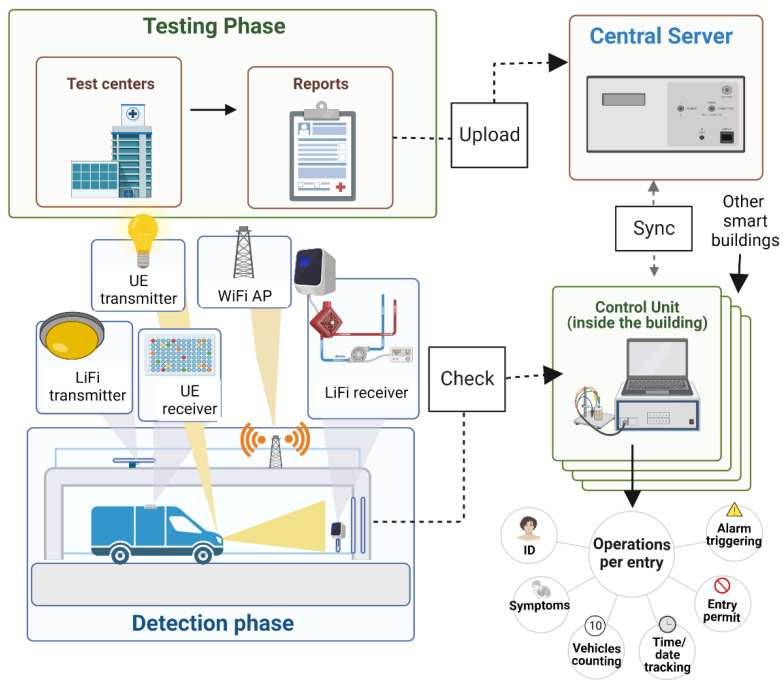
Illustrations of the proposed real-time monitoring, vehicle tracking, and scheduling system for smart buildings.

In this section, the social distancing approach for limiting the number of vehicles, SDA-LNV, is introduced and discussed including the LiFi system, system setup, and components. Moreover, the proposed SDA-LNV algorithm and the working principle are discussed. A LiFi transmitter (LF-Tx) and receiver (LF-Rx) are placed at the building entrance, where the LF-Rx is fixed on the gate facing outwards and the LF-Tx is fixed on the ceiling above the car facing downwards. On the other side, a user equipment (UE) which is installed in the vehicle has a transmitter and receiver as well, the UE’s transmitter (UE-Tx) and UE’s receiver (UE-Rx), respectively. The UE-Tx is fixed at the front of the car with the lights, and the UE-Rx is fixed at the car roof facing upwards. Some LiFi systems that consider uplink (UL) and downlink (DL) design use VL for the DL and different mediums for the UL such as infrared or WiFi in order to avoid interfering with the VL that comes from the DL such as in the case of using cell phones [18]. However, in this system design, VL is used for both since the UL and DL transmissions are not adjacent. The control unit (CU) monitors everything from inside the building and records all entries and exits and is responsible for making operation decisions according to the predefined rules. In addition, the central server (CS) is a high-speed cloud server [44] connecting all testing centers with all smart buildings. All components of the system model are illustrated in Figure 4.

It can be seen from the above diagram that the overall system design consists of two main operational phases. Each phase includes its own set of tasks. The phases and their activities are covered in more detail below. The suggested design has various requirements. First of all, system components included with the UE in the vehicles that have access to the smart building have to be installed and training must be provided to the drivers. The two phases are explained as follows:

I. **Testing phase (Data Collection):** In this phase, test sites will collect primary data on COVID-19 participants. When a person enters a COVID-19 test facility, basic data such as residence, employment, car plate number, and active mobile phone number(s) should be collected. The test result of COVID-19 with the statistics documented beforehand will be reported to the central database (negative or positive).
II. **Detection phase (Vehicle entry):** This phase consists of a few steps including vehicle detection where the access point assignment (APA) process takes place. If the LiFi AP is not available for any reason, the AP will be transferred or assigned to WiFi AP. In general, when the vehicle approaches the building’s gate of entry, the receivers of both LiFi and UE are in listening mode. At this point, UE-Rx detects LF-Tx signals and receives instructions when a vehicle approaches; a message with instructions will appear on the phone screen of the driver in the vehicle asking the driver to proceed to the next step (using the phone app connects UE-Rx and UE-Tx with BLE. The instruction consists of a request for transmitting information and requests entry access (this process is equal to card touching in normal systems). The concept of transmitting data from UE-Tx to LF-Rx could be similar to the concept in [45]. The transmitted information will be verified and analyzed in real-time by the CU with the help of the CS (note that all drivers are assumed to be tested every week). Then, the CU checks the number of vehicles inside the building, symptoms, and the test results obtained from the test centers via the CS; if there is a violation (such as the vehicle count inside the building has reached its maximum, or the driver is susceptible/infected), an alert is triggered, and new instructions are given. The allowed number of vehicles inside the building differs from one place to another based on the size and capacity of the building; this value is to be set at the time of the system setup. To lower the burden on the CS, the CU of each building syncs data with the cloud server at the CS once per day. When no violation occurs, entry is granted for the vehicle (V++) and when the vehicle exits the building the same process can be done for the vehicle count (V−−). If the maximum count of vehicles is achieved, the system shall prohibit access and advise the passenger to come back later (the period of waiting time can be specified based on capacity and some other factors based on the operations and processing inside the building).

During each entry, the CU will make a few operations such as identification (ID) check, symptoms check, vehicle counting, time and date tracking, entry permit or deny, and alert triggering. It should be highlighted that depending on a centralized repository potentially leads to a variety of security attacks such as man in the middle (MITM), distributed denial of service (DDoS), structured query language (SQL) injection, and so on. These attacks, unfortunately, are typical for centralized databases. We suggest employing cutting-edge security mechanisms to protect against potential threats. Privacy considerations are addressed from the perspectives of carriers, small enterprises, users of the proposed model, and non-users. Users’ privacy will not be compromised since all private details will not be made public. Furthermore, the system will not demand location data. The proposed model is intended to function in such a way that the data of users is stored before the COVID-19 test for tracking and evaluation. As a consequence, it is not required to mask the carrier’s identity later on. As a result, no special permission will be necessary for using their data in the system since it is a casual and standard procedure that does not include any sensitive information such as location tracking, etc. However, because the system involves alert triggering and informing authorities, the proposed model demands the users’ approval. The proposed model’s adoption by local companies is mainly influenced by government policies. Throughout the enrollment process, the user will be obligated to provide their mobile phone number as well as their vehicle plate information. The proposed design, which will be placed in a smart building, is anticipated to greatly decrease infection risks.

LiFi is a bidirectional, high-speed, entirely networked wireless connectivity technology that uses visible light for lighting and transfer [17]. LiFi is basically a light fidelity that functions in a similar way to WiFi, but uses a VL medium of transmission and is 100–1000 times greater in data rates. In terms of bandwidth, reliability, and capacity, LiFi technology has also advanced in that it also consists of both a transmitter and a receiver. An RF chain transforms modulated electrical signals into RF electromagnetic pulses. In LiFi networks, transmitter front-end parts transform a modulated electrical output to a light signal, while receiver front-end equipment transforms the returning optical pulses into an electrical signal. Many low-cost fragmented LiFi front-ends utilize intensity modulation (IM) with direct detection (DD) for downlink transmission. IM/DD modifies downward transmission route characteristics [46].

**Figure 5:**
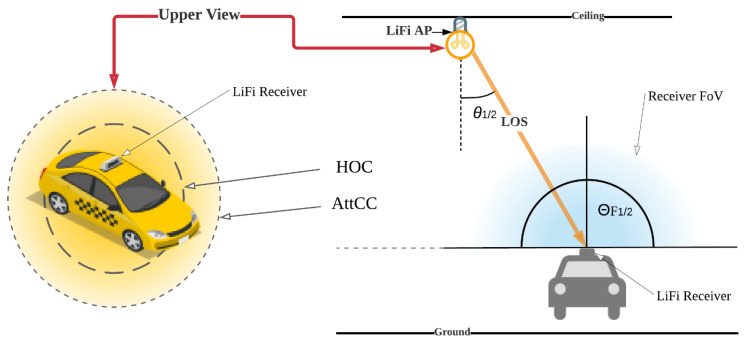
Illustrations of the HOC inside the AttCC.

A LiFi AP mainly aids an inadequate region of around 2–3 m in radius, which fulfils the space and coverage area requirement for the vehicles at the confined area of the vehicle entrance pathway. Since the users are in mobile movement, it is necessary to consider the mobility issue. In addition, it is important to consider multiple access points where the building could have more than one gate. In this case, coordinating and balancing the load is important for increased reliability. In a homogeneous network, overlapping LiFi AP coverage must be reduced to minimize inter-cell interference; otherwise, the LB procedure is needed. In homogenous networks, the signal strength strategy (SSS) puts each user with the AP with the strongest signal strength. “Attocell” is the LiFi AP’s coverage area. The configuration of LiFi APs affects the LiFi operational area geometry in optical co-channel interference (CCI) conditions [47]. Figure 5 illustrates the handover circle (HOC), a high-gain service region, and the attocell circle (AttCC), a low-gain crucial sector.

CCI decreases LiFi’s spectrum efficiency whenever APs are near. If LiFi APs are not greater than an attocell distant, an efficient solution should be used. Users connect within a LiFi attocell’s service region. Attocell size and illumination are affected by the range between the AP transmitter and receiver, ceiling height, and optical energy transfer. LiFi AP users must reside inside HO circles for a steady connection. Non-CCI LiFi AP users will receive optical signals from one LiFi APs. Each LiFi AP serves the same-sized attocell. To avoid optical interference, LiFi APs should be farther apart than attocells.

Light Emitting Diode (LED) lamp brightness must fulfil interior illumination and photobiological health requirements [48,49]. A standard LED chip yields a few mW of optical energy, significantly less than everyday illumination needs. To create a lamp with thousands of lumens, it is necessary to integrate multiple small LED chips to create an LED array, which is equivalent to a single high-energy light source. Most high-powered LED lights employ this method [50].

In order to function properly, it is necessary to take into account a wide range of specific characteristics, such as colour presentation, unified glare grade, lux level, uniformity, and so on. These variables must remain within the limit. All of these measures are not important in a communication system; hence, they are not analyzed here. This necessitates the use of a straightforward technique for estimating the lamp’s luminous efficacy. The lamp’s optical output specifies the maximum signal energy, while the transmitter’s radiation parameters decide the distribution area, attocell area, and irradiance angle φ. 

A dynamic load balancing technique is proposed in this research approach to control and coordinate the connection among APs and vehicles to satisfy specific per-user Quality of service (QoS) including processing time, delay, and decision process while maintaining minimum outage performance. For both downlink and uplink, a standard LiFi system is adopted. Thoroughly specified are the system and channel models, protocol, and equations.

The LiFi network with multiple APs is considered. *N_v_* represents the quantity of LiFi APs. All mobile vehicle photon detectors (PD) face the ground (this means the angle of irradiation is equal to that of incidence). Furthermore, because each vehicle entrance consists of a single LiFi AP, CCI with other LiFi AP is prevented. Therefore, this study ignores CCI. Each LiFi AP covers a limited cell. This network’s users are randomly distributed between AP gates. 

Due to the ability of LiFi APs to repurpose previously unused capacity, the LiFi system has the potential to offer consumers high spatial-spectral effectiveness. Mobile users’ optical channel state information (CSI) differs by coverage area, and optical signal gain could be weak when rays of light are blocked. For this issue, a WiFi network is added to the system to boost user data rate capability and to overcome the blockages of LiFi line of sight LOS. When a connected LiFi user receives no optical gain caused by blockages or disfunction of any entity, the user is relocated to the WiFi AP. This event is termed as handover (HO), and when it occurs within different wireless technology, such as WiFi and LiFi, or vice versa, is considered as vertical handover (VHO). These frequent HOs increase the chance of connection interruptions, packet losses, delays, and a poor user impression. Since LiFi uses different electromagnetic spectrums, there is no interference between LiFi and WiFi.

Hybrid LiFi and WiFi networks were created to combine LiFi’s fast data rate with WiFi’s widespread coverage [18]. VHO can be built employing whatever mathematical technique fits a multiple-input, single-output function. VHO procedure components include metric gathering, decision-making, and HO action. After adopting a suitable mathematical instrument to represent VHO, the algorithm’s performance must be tested. In homogenous networks, SSS places every user with the AP with the strongest signal.

In this study, the number of LiFi AP is assumed to be four, and WiFi AP is four. Since receivers are mobile, the CSI of combined LiFi and WiFi lines changes, demanding regular resource allocation.

When using a traditional LiFi network [51,52,53], user CSI changes slightly, meaning it lasts briefly, because a single LiFi AP might serve differently in a dynamic environment, specifically after a HO occurs. This would cause two issues: (i) delay caused by the HO between LiFi and WiFi, and (ii) since WiFi has a wide range of transmission, it is possible for users that are waiting in the queue at the entrance to connect to the AP even before reaching the entrance. To solve these issues, the proposed system design, a single user (the vehicle) is designed to connect LiFi AP first before trying to pair with the WiFi, where the user will be exposed to a single LiFi AP where other users have no probability of connecting the targeted LiFi AP nor the WiFi AP. Driven from that perspective, and to avoid the above two issues, the user requests access using LiFi AP; then, in case of trouble, the system asks the user whether to start the WiFi pairing. CU should monitor the system at frame intervals. All users get allocation results from the CU and AP signals at consistent data rates during T*p*. The state design numeral is *n*. Users who are able to benefit from a LiFi data rate of more than γ are allocated to LiFi APs, while those who are not are assigned to WiFi APs.

An averaged data rate for each user is denoted as Γ. Here, *N_u_* is the number of the users; *N_s_* is the number of the working states; *C* ℒ = {*v*|*v* ∈ [1, *N_v_*], *v* ∈ Z} is the set of optical attocells; and *C Ʀ* = {*r*|*r* ∈ [1, *Nr*], *r* ∈ Z} is the set of WiFi cells. An HO occurs in case of a broken, damaged, or dirty receiver, and/or unfunctional UE LiFi transmitter.

After proper modulation [54,55,56] on the transmitted side, the data will be forwarded in binary mode via LED bulbs. The binary signal is applied to calculate the pulses. Logic one represents high and logic zero denotes low. The PD is utilized on the receiver side to receive the logic pulses sent by the LEDs, which are then used to enhance the signal and provide the output [18]. The optical channel gain of a line of sight (LoS) channel of LiFi is defined as:

(2) $$H=\left\{\begin{array}{l} \frac{\left(\left(m+1\right)A_{p}\right)}{2\pi (〖{z〗}^{2}+h^{2})}g\left(\theta \right)Ts\left(\theta \right)\cos^{m}\left(\phi \right)\cos\left(\theta \right),\;\;\;\theta <\Theta_{F}\;,\\ 0,\;\;\;\;\;\;\;\;\;\;\;\;\;\;\;\;\;\;\;\;\;\;\;\;\;\;\;\;\;\;\;\;\;\;\;\;\;\;\;\;\;\;\;\;\;\;\;\;\;\;\;\;\;\;\;\;\;\;\;\theta >\Theta_{F}\;, \end{array}\;\;\;\;\right.$$

where *m* is the Lambertian index, which is a parameter of the half-intensity emission angle *θ*_1/2_, given as *m* = −1/log 2(*cos*(*θ*_1/2_)); *z* is the horizontal distance from a LiFi AP to the optical receiver, *Ap* is the receiver’s physical area of the photo-diode, *h* is the height of the room, Θ*_F_* is the half angle of the receiver’s FOV, *Ts*(*θ*) is the gain of the optical filter, *ϕ* is the angle of irradiance, *θ* is the angle of incidence, and g(*θ*) is the concentrator gain:

(3) $$g\left(\theta \right)=\left\{\begin{array}{c} \;\frac{x^{2}}{sin^{2}\Theta_{F}},\;\;\;\;\;\;\;\;\;\;\;\;\;\;\;\;\;\;\;\;\;\;\;\;\;\;\;0\leq \;\theta <\Theta_{F}\;\\ 0,\;\;\;\;\;\;\;\;\;\;\;\;\;\;\;\;\;\;\;\;\;\;\;\;\;\;\;\;\;\;\;\;\;\;\;\;\;\theta >\Theta_{F}\; \end{array},\;\;\;\;\right.$$

where *x* is the refractive index. The LED bulbs in a LiFi system function in the direct area, where the output optical energy is proportionate to the input voltage. Modulated electric signals are bias-voltage before LiFi transmission. IM/DD ensures that only real-valued signals are delivered to recipients. The next formula governs the conversion of the median electric energy of signals to average optical energy:

(4) ı=PoptPt

where *P_t_* is the electric power of the signals, and *P*_opt_ is the regular transmitted optical power of LiFi AP which is relative to ϰ_DC_; for an assumed user *μ* associated with a LiFi AP *α*, the Signal-To-Interference-Plus-Noise Ratio (SINR) can be written as:

(5) $$SINR_{\mu ,\alpha }=\frac{{\left(\kappa P_{opt}H_{\mu ,\alpha }\;\right)}^{2}}{\iota^{2}N_{0}B+\sum {\left(\kappa P_{opt}H_{\mu ,else}\;\right)}^{2}}\;,\;\;\;\;\;\;\;\;\;\;\;$$

where the noise power spectral density is *N_0_* [A2/Hz], *H* (*μ*, *α*) is the channel gain between user *μ* and LiFi AP, and *H* (*μ*, *else*) is the channel gain between user *μ* and the interfering LiFi AP. K denotes the efficiency of optical to electric conversion at the receivers. At least half of the sub-carriers should be used after modulation to recognize the complex-valued sign’s Hermitian conjugate. In state *n*, signal delivery uses just half of the available bandwidth. The Shannon capacity is used to compute the achievable data rate between both the user *μ* and the LiFi AP *α*, which is stated as:

(6) $$R_{\mu ,\alpha }^{\left(n\right)}=\frac{B_{L}}{2}\;\log_{2}(1+SINR_{\mu ,\alpha }^{\left(n\right)}),$$

where $B_{L}$ is the bandwidth for optical signal broadcast. The time division multi-access (TDMA) method is used in this exertion, and a proportional decent scheduler is studied. Once proportionate equality is detected, LiFi AP users have equal time. Low energy from mirrored routes flattens the channel’s harmonic responsiveness, so all sub-carriers allocated to a user adopt the identical CSI. RF cell WiFi APs have omnidirectional broadcast channels. In RF, orthogonal frequency-division multiple access (OFDMA) is employed. The WiFi channel gain across users and WiFi APs is estimated as follows:

(7) $$h=\sqrt{10\frac{-L\left(d\right)}{10}\;\;}\left(\sqrt{\frac{K}{1+K}}h_{d}+\sqrt{\frac{1}{1+K}}h_{s}\right),$$

where *h_d_* = $\sqrt{1/2}\left(1+j\right)$ is the straight lane declining channel; *K* = 10 dB is the Rician component for internal 60 GHz connections; *h_s__~_C Ɲ* (0,1) is the scattered path fading channel; *L*(*d*) is the corresponding large-scale fading loss in decibels at the isolation range *d*, given as:

(8) $$L\left(d\right)=L(d_{0}+10\nu \;\log_{10}\left(d/d_{0}\right)+X,$$

where *L*(*d*_0_) = 68 dB is the benchmark path loss at *d*_0_ = 1 m; *X* is the shadowing factor, which is considered to be a zero mean Gaussian distributed arbitrary variable with a standard deviation of 1.8 dB, and *v* = 1.6 is the route loss exponent; the shadowing impact produced by human figures adjacent to the mmWave radio acquaintances is overlooked. Each user can be granted sub-carriers with flexible power. Data rate gain due to WiFi connectivity between user *μ* and WiFi AP α in state *n* can be written as:

(9) $${ϒ}_{\mu ,\alpha }^{\left(n\right)}=B_{\mu }log_{2}\left(1+\frac{⎡h_{\mu ,\alpha }^{\left(n\right)}⎤{}^{2}P_{R}}{N_{0}B_{R}}\right)$$

wherein $h_{\mu ,\;\alpha \;}^{\left(n\right)}$ is the WiFi channel gain diagonally the user *μ* and the AP α according to (6), and *B_μ_* is the bandwidth specified to the subscriber *μ* in the WiFi system. Λμ is labelled as the proportion of bandwidth gained by the user *μ*. As a result, *B_μ_* can be written as:

(10) $$B_{\mu }=\Lambda_{\mu }B_{R.}$$

An HO matures in an active system as soon as two separate APs support a user in two adjacent states. There are no substantial data acknowledged by the participants throughout the HO. These origins, overhead (OH) and spectral effectiveness impairments, are taken into consideration in this analysis. The OH of numerous types of HOs are measured in milliseconds (ms). This OH is hypothetical to be pointedly fewer than the chronological interval *Tp* from the two states. It is thought that the Poisson distribution is an appropriate model to describe it. As a result, the OH of various types of HOs is demonstrated in this study as an accidental flexible with an autonomous identical Poisson distribution. The probability mass function can be calculated as follows:

(11) $$\mathrm{Pr}\left(t_{ij}=x\right)=\frac{\zeta_{ij}^{x}e^{-\zeta \mathrm{ij}}}{x!},x=1,2,3\ldots$$

where ζ_*ij*_ = E[*t*_*ij*_] is the OH mean, *t*_*ij*_ is the OH of the AP shift from AP *i* to AP *j*, and x is the period interval of OH measured in (ms). The transmission effectiveness between two neighbouring states can be expressed as follows:

(12) $$\eta_{ij}=\left\{\begin{array}{l} 1-\left[\frac{t_{ij}}{T_{p}}\right]\;\;\;\;\;\;\;\;\;\;\;\;\;\;\;\;\;i\neq j,i,j\in C_{L}\cup C_{R.\;}\\ 1,\;\;\;\;\;\;\;\;\;\;\;\;\;\;\;\;\;\;\;\;\;\;i-j \end{array}\right.$$

where the procedure [.]^+^ stands for maximum (., 0). The produce of efficiency in (10) and the interaction link data rate yields the impactful data rate with HO between each AP and user. During the working period, users move in a random manner in the indoor scenario, and the APs assigned to them are changed based on their position and status. The CU computes the allocation result in each state while taking HO into account. Algorithm 1 depicts the evolving algorithm implemented by the CU in *N_s_* working states. After the determination of AP selection and LB, the proposed algorithm performs data swapping and comparison, including ID checking, COVID-19 test results, vehicle counting, and entry decision.

The serving AP in state *n* − 1 for user *μ* is signified as α′*_μ_*. To wholly utilize LiFi’s spatial-spectral efficiency, users would be assigned to LiFi APs first in each state. Users who obtain decreased data rates than the threshold γ, are re-allocated to WiFi APs using the data rate threshold. The very first distribution phase employs a criterion of maximum effective throughput. The LiFi AP obtaining the greatest connectivity data rate with HO can be expressed as follows for users:

(13) $$\beta_{1,\mu }=\arg\;\max\;\;\;\;\;\;\eta_{\alpha^{\prime }}{}_{\mu }{\;}_{j}R_{\mu ,j}.\;\;\;\;\;\;\;\;\;\;j\in C_{L}$$

Here, $\eta {\alpha^{\prime }}_{\mu }j$ is the transmission efficiency of the linked LiFi user in the following state *n*, $\beta_{1,\mu }$ is LiFi AP with the highest communication link data rate with HO, and $R_{\mu ,j}$ is the attainable LiFi data rate in the next state. The optical data rate for every user can be composed as:

(14) $$\Omega_{\mu }=\eta_{\alpha^{\prime }}{}_{\mu }\beta_{1,\mu }\;\;\;\frac{R_{\mu ,\beta_{1,\mu }}}{M_{\beta_{1,\mu }}},$$

Here, *M_β_*_1,*μ*_ represents the number of users supplied by LiFi AP *β*_1,*μ*_. Users who meet the condition $\Omega_{\mu }$ < γ are re-allocated to WiFi APs during the re-allocation phase. To meet the average data rate requirement (ADRR), the data rates of users in the WiFi system should therefore be improved. In a nutshell, the current CSI is used to determine the optimal WiFi AP for each user. Allocating APs in the WiFi system follows the same maximization of useful throughput criterion as the LiFi system. The best WiFi AP for the user *μ* is displayed as:

(15) $$\beta_{2,\mu }=\arg\max\;\;\;\;\eta_{\alpha^{\prime }}{}_{\mu }{\;}_{j}{ϒ}_{\mu ,j}\;\;\;\;\;\;\;\;\;\;\;\;\;\;\;\;\Omega_{\mu }<\gamma .\;\;\;\;\;\;\;\;\;\;\;j\in \;C_{R}.$$

where the arg max probability distribution locates the maximum random variable among a set of random variables, and ${ϒ}_{\mu ,j}$ is the achieved WiFi data rate for a user *μ* in the next state *n*. In addition, the average data rate attained in earlier stages is taken into consideration when assigning bandwidth dynamically. The bandwidth percentage Λ*μ* can be set up as follows to increase the number of users who previously had a low typical data rate:

(16) $$\Lambda_{\mu }=\frac{1/\lambda_{\mu }}{\sum \;{\;}_{x\in U_{R}{}^{1/\lambda_{x}}}}$$

where $U_{R}$ is the collection of users supplied by WiFi APs in the present state, and λ*_μ_* is the median data rate for subscriber *μ* in the prior states. This kind of bandwidth allocation can boost the transfer rates of some users. As per (12) and (14), the AP assigned to subscriber *μ* in state *n* can be inscribed as follows:

(17) $$\alpha_{\mu }=\left\{\begin{array}{c} \beta_{1,\mu ,\;\;\;\;\;\;\Omega \;\geq \gamma }\;\\ \beta_{2,\mu ,\;\;\;\;\;\;\Omega \;<\gamma }\; \end{array}\right.$$

Thus, the user’s achievable data rate can be proved as:

(18) $$r_{\mu }^{\left(n\right)}=\left\{\begin{array}{c} \;\eta_{\alpha_{\mu \;\;\;}^{\prime }\alpha_{\mu }}\;\frac{R_{\mu ,\alpha_{\mu }}}{M_{\alpha_{\mu }}},\;\;\;\;\;\;\;\;\;\;\;\;\;\alpha \in \;\;C_{R},\;\;\;\;\;\;\;\;\;\;\;\;\;\\ \eta_{\alpha_{\mu \;\;\;}^{\prime }\alpha_{\mu }}\;\Lambda_{\mu }\Upsilon_{\mu },\alpha_{\mu }\;\;\;\;\;\;\;\;\;\alpha \in \;\;C_{R}\;\;\;\;\;\;\;\;\;\;\;\;\;\;\; \end{array},\right.$$

where *M_α_μ__* represents the number of users provided by the LiFi AP α*_μ_*. The outage probability of the QoS requirement in the hybrid system can be confirmed using the ADRR Γ as:

(19) $$\Delta =Pr\left(\frac{1}{N_{s}}\;\overset{N_{8}}{\underset{n=1}{\sum }}r_{\mu }^{\left(n\right)}<\Gamma \right).$$

where $N_{s}$ is the sum of occupied states. Giving to the presented dynamic LB algorithm, the data rate criterion can have a significant influence on the likelihood of an outage. For θ_½_ = 90° (perpendicular surface PS), the peak theoretical optical gain is obtained. As a result, the optic must be in significant contact with the chip. The receiver gain is a useful metric for categorizing the optic module’s operation. For the best possible dynamic range, the gain should be consistent across the field of view (FOV).

Note that all the equations above (2)–(19) are considered for the WiFi and LiFi connection including transmission, APA, transmission evaluation, HO process, load balancing, data rate evaluation and calculations, etc., which were all introduced in our previous study [57]; the rest of the processes and steps for our proposed system are introduced in Algorithm 1. The following are some highlights of the procedure preceded by Algorithm 1 including APA, vehicle entry decision, and monitoring, as explained below. When the system starts, SDA-LNV begins. Establishing a workable setup for the system, turning on all essential components, and identifying all accessible access points are key duties. The proposed algorithm consists of many steps, which can be summarized as follows:

I. **First**: variables are set and created, such as *Vnew*, which represents every new user to the system. *Vin* represents the number of vehicles inside the building while *Vout* are the ones exiting the building; *Vsh* represents the threshold of the allowed number of vehicles inside the building at a time. The variables *V_lifi* and *V_wifi* represent the vehicles connected to the LiFi AP and WiFi AP, respectively. *P_ptv* and *P_ntv* represent the users that were marked as positively infected with COVID and not infected, while *P_wtg* means the user is marked as waiting their turn for entry, and *P_den* represents those who were denied entry. The term *TR* signifies the test results, where *TR_μ_* points to the test results of a specific user.
II. **Second**: the status of all sensors and APs is checked at the start of the system and the CSI of all connected users is obtained. When a new user enters the coverage area and sends an entry request from the UE-Tx to the LF-Rx, the system starts the APA process where the vehicle would connect to the LiFi AP or the WiFi AP. If the optical gain of the LiFi receiver UE-Rx receives a data rate higher than a certain data rate threshold from the transmitter LF-Tx, the user will be connected to the LiFi; otherwise, it will be transferred to the WiFi AP.

**Algorithm 1** The proposed SDA-LNV algorithm performed via the CU1.**Booting:** set variables, Vnew; Vin = 0; Vout = 0; Vsh = 10; V_lifi; V_wifi; P_ptv; P_ntv; P_wtg; P_den; TRμ, TR_ptv; TR_ntv; η; ϒ; R; H; h; Ω;//monitoring 2.Whereas ***n*** ≤ ***Ns*** do3.Obtain CSI for all connected users and APs;4.If all vehicles’ CSI are obtained, then5.Calculate R, ϒ*,*   η, and H among all vehicle and APs;//Start APA process and decisions 6.For new vehicle *Vnew* do7.Calculate $h,\;\Omega ,\;,\beta_{1,\mu }\;,\beta_{2,\mu }$8.If (${Ω}_{\mu }$ ≥ γ)9.User *μ* is assigned to LiFi AP *β_1,μ_*10.*V_lifi++*;11.Else12.User *μ* is assigned to WiFi AP *β_2,μ_*13.*V_wifi++;*14.*Compute achievable bandwidth *B_L_ and Λ_μ_** //Check (1) driver test results
15.If ($\;TR_{{\;}_{\mu ,\alpha }}^{\left(n\right)},$ == *TR_ptv*)16.**Access denied**: *P_ptv = ++P_den;*
//Check (2) vehicle numbers inside the building17.Else if (*Vin ≥ Vsh)*18.**Go to the waiting area**: *P_ntv = ++P_wtg;*19.Else20.**Access granted**: *Vsh* = *Vsh+1, Vin*++, *P_ntv++;*21.Calculate *Vin, Vout, V_lifi, V_wifi, P_wtg, P_ntv, P_ptv, P_den;*22.End if23.End if24.End if25.Compute the average data rate per user $\lambda_{\mu }$26.Compute the achievable data rate in all the states $r_{\mu }^{\left(n\right)}$27.End for28.Else29.Refresh and update the CSI, C*ser*;30.End if31.Update AP α′*_μ_* in the current state32.n ← n+1;33.End while

**Figure 6:**
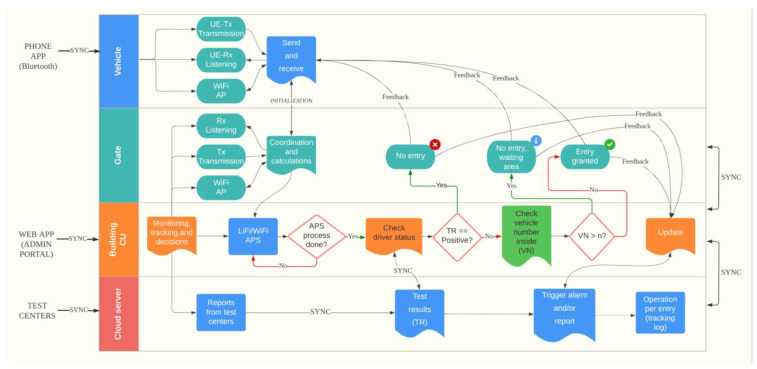
System model process flow diagram.

**Figure 7:**
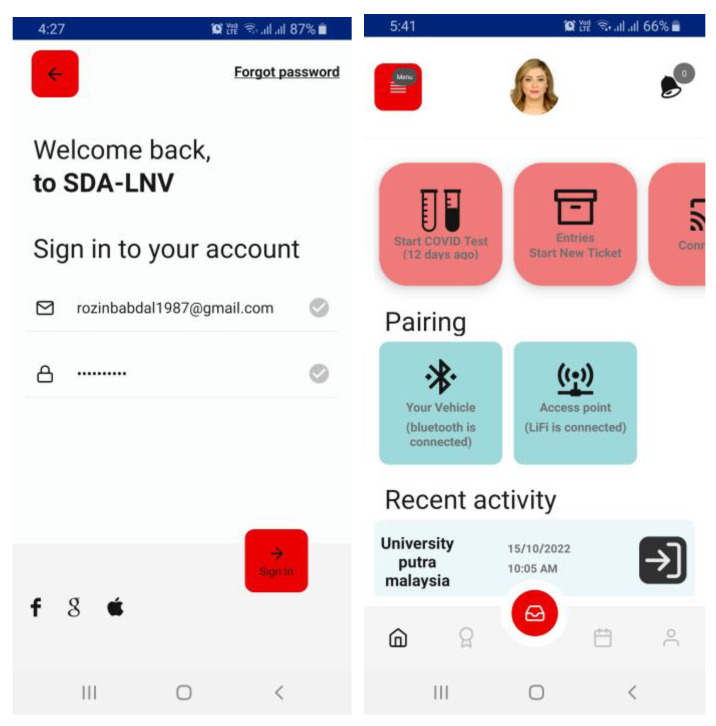
The suggested phone-app design.

Figure 6 represents the step-by-step process of the process flow which consists of four main sections, explained as follows:

**Vehicle side:** The vehicle represents the user in this dynamic system. All the sensors attached to this entity including LiFi and WiFi APs are connected to the driver through a smartphone application using BLE, which provides the driver with a few options, including the ability to initiate and send an entry request upon reaching the gate, check his COVID test results, check the connection status, check the entries history, and more. The vehicle is supposed to be connected to the building system once it is paired with the APs of the smart building, and when the vehicle leaves the area of service it will be disconnected from the system. The user is responsible for sending and receiving information from and to the vehicle and the gate of the entrance after making sure the devices are paired. The driver will be notified of any update on his medical status in addition to the entry request where the system will determine whether the user is allowed to enter, wait for a while, or be denied. The driver will be able to contact the building admin through the app as well. Check Figure 7 for the suggested phone-app design.

**Gate side:** This is an important entity that is responsible for sending and receiving data from and to the nodes of users and the system. The collection of data at high speed will provide high performance and low latency for decision-making. All entities installed at the gate connect the user to the control unit at the building for easy monitoring and tracking. Any event related to entries and access point assignments is to be sent as feedback to the user and to the CU.

**Figure 8:**
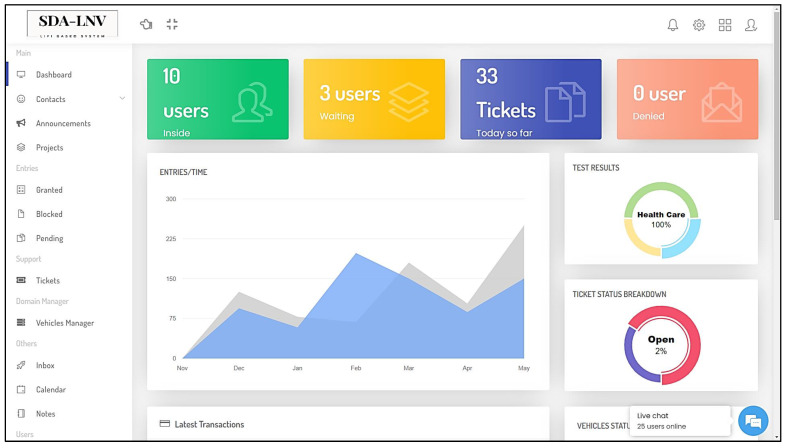
Web-app portal design for the admin of the building.

**Building side:** The monitoring and tracking processes begin at this unit plus the control and coordination of vehicles’ entry, support, announcements, and more. The administrator has access to all features including system files, user accounts, and supervision. Moreover, the APA process for the users is executed at this unit with the help of the APs at the gate and the cloud server. The number of vehicles inside and outside the building is calculated based on the status of users inside the building, according to their connection to the APs which is measured by the data rates achieved through the service area. Furthermore, the system checks the COVID test results before making the decision by making a regular connection to the cloud server where the health examination results are saved for all drivers. All commands are sent from the CU to the gate side and the main server for tracking logs. An alarm is triggered and the authorities will be notified in case of detection of a positive case at the time of entry request and/or receiving test results. Figure 8 shows the web-app portal design for the admin of the building.

**Cloud server side:** The CU makes a decision with the help of the cloud server. The patient data are stored in the cloud repository effectively. The patient needs to provide a username and password to access their data. The critical aspect of cloud storage is the ability for patients to synchronize their data to the medical database, which can hold a full copy of the data from the remote server. Any updates to client status are synced between the cloud medical database and cloud repositories. The cloud can hold static and dynamic patient data. No one but the patient may access the cloud repository’s patient data due to adequate authentication and authorization. Data are encrypted while stored in the cloud and decrypted when accessed. The government, healthcare agencies, and doctors get all patient information so they can control the COVID-19 epidemic. Doctors and researchers can use this data to study and treat patients.

## 4. Results and Discussion

Here, we show the experimental results from testing the proposed system model and our novel algorithm. To test variations of our SDA-LNV algorithm, we use the simulation capabilities of the MATLAB programme. This section details the scenario and system configuration that will be used for modelling. Next, the results of the simulations are discussed. Future research could focus on developing a system that can operate in real-time, similar to the one proposed. To do this, the proposed algorithm will be put into place, which will decrease the infection rate, boost the system’s performance in terms of wireless communication speed, and direct users to the best APs in the coverage area.

This work’s performance evaluation can be broken down into two sections: the first is concerned with the hybrid LiFi/WiFi network and covers topics such as the HO and APA of networks, throughput achieved by the system, fairness, average data rates, and vehicle response to the system over time. Vehicle waiting time (queue time), detection rate, and replica ratio all factor into the second section, which discusses social distancing efficiency.

**Figure 9:**
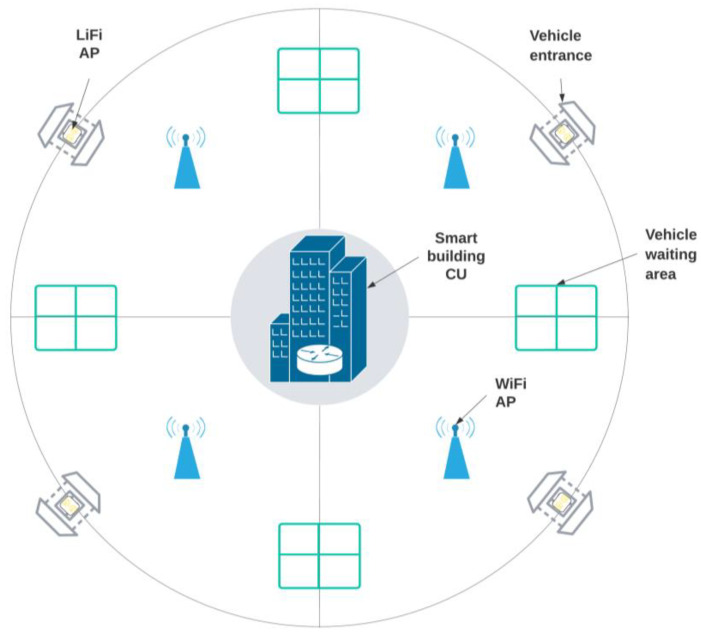
Simulation scenario.

By incorporating LiFi technology into this SD system, we have increased the system throughput that is visible to the user as the service provided by the network, allowing for superior wireless communication in comparison to other wireless devices. In order to fully grasp the significance of our proposed system, we will compare it to previously investigated, state-of-the-art wireless technologies for SD. These include WiFi and BLE. Each door has two LiFi APs installed in it. The building has four designated entry points, requiring four LiFi APs. Since WiFi is only used as a complementary communication, four APs are used in this scenario, and they are all evenly spaced so that each AP is located near gates. In addition, VHO occurs as a result of an automatic handoff from the vehicle to the WiFi AP after permission is granted. The simulation layout is shown in Figure 9. The simulation’s primary parameters are listed in Table 3.

**Figure 10:**
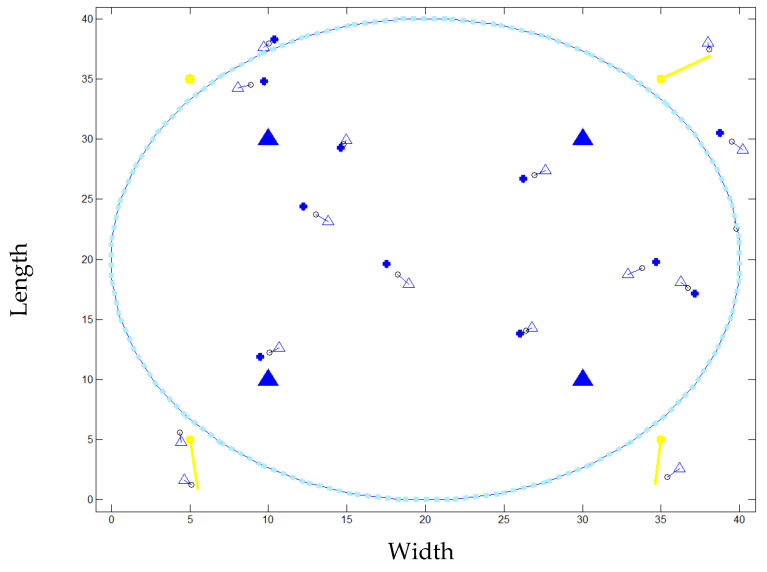
Simulation execution of the proposed model.

The CCI between neighbouring LiFi attocells is ignored and the same amount of bandwidth is reused by all of the LiFi APs. All the vehicles move around at random speeds, which can be anywhere from zero to few meters per second. When a single user is given access to two APs in neighbouring states, this is called an HO. Poisson distribution in all directions characterizes the HO overhead. However, in a number of studies, the HO overhead was factored in. Even if the user crossed the HO circle or satisfied a triggering condition such as optical gain, HO overhead would prevent the user from being relocated to another AP. There are a number of factors, including resource allocation and data rate requirements, that can add delay to the HO process when determining an APA. Hybrid networks necessitate a consistent data transfer rate among their users. Figure 10 shows the proposed model in action with 15 simulated objects.

**Figure 11:**
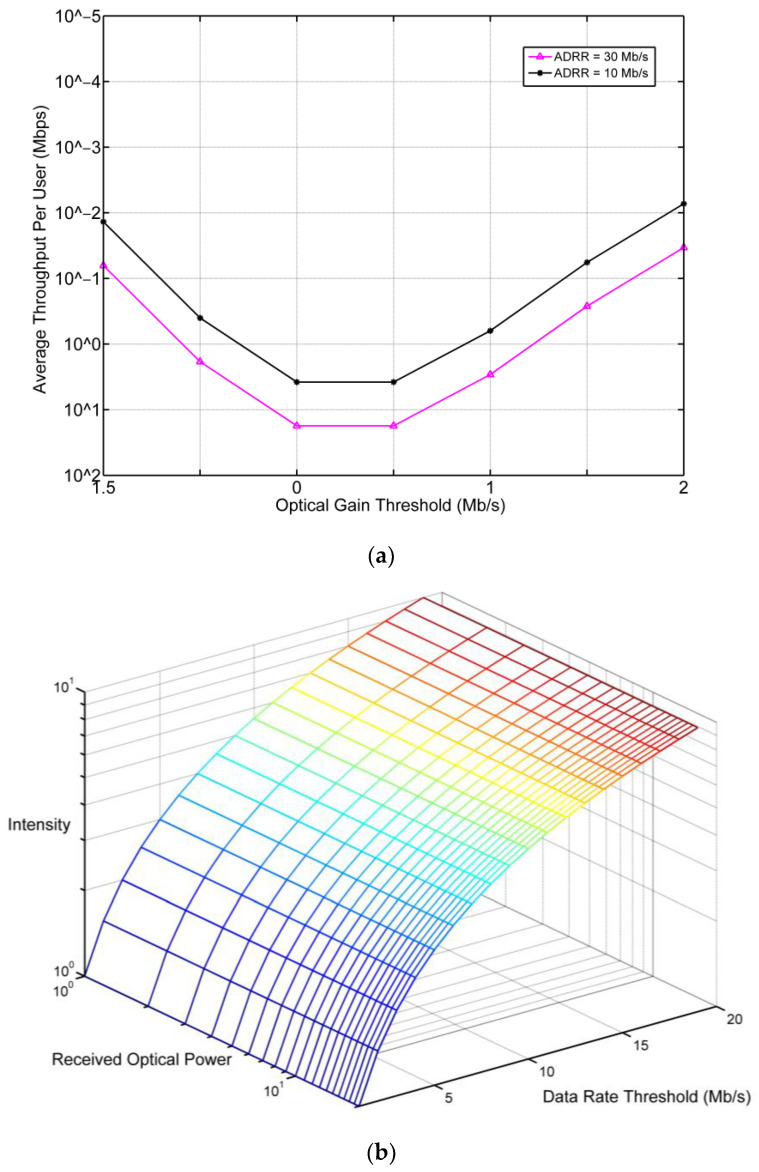
HO probability correspondence with the optical gain threshold: (**a**) the HO*p* corresponding to the optical gain threshold γ with the consideration of different ADRR values, and (**b**) optical power correspondence with intensity and the optical gain threshold.

To examine the HO between the LiFi AP and WiFi AP at the entrance at the time of APA for vehicles, fair measurements of HO probability (HO*_p_*) corresponding with the optical gain threshold γ with ADRR) values are shown in Figure 11. Figure 11a shows the HO*_p_* corresponding to the optical gain threshold γ with the consideration of different ADRR values for the user and the received optical power with the intensity value. The value of the γ is represented as data rate measurement. As shown, the HO*_p_* is a convex function corresponding to different values of data rates and is impacted by the ADRR. The HO*_p_* increases with the increase of the ADRR, where it reaches a maximum of 30 Mbps of ADRR and a minimum of 10Mbps. It can be seen that when γ = 0, the HO*_p_* reaches the peak negative for all ADRR values which means an HO is triggered whatever the ADRR values are. This is because when the user receives low optical gain, the condition of the HO and APA is satisfied (lines 8, 9, and 12 in the SDA-LNV algorithm). Note that the positive value of HO*_p_* means a higher chance of HO occurrence and vice versa. With the increase in ADRR, the value of the HO*_p_* becomes critical when the value of γ ranges from 0 to 1, where 30 Mbps of ADRR results in positive HO*_p_*, whereas 10Mbps leads to negative HO*_p_* at some point. This is because low optical gain does not satisfy the expected QoS for the user. Moreover, when γ > 1.5, the HO*_p_* remains negative and that means no HO is expected. On the other hand, Figure 11b illustrates the correspondence of received optical power to the γ and intensity.

**Figure 12:**
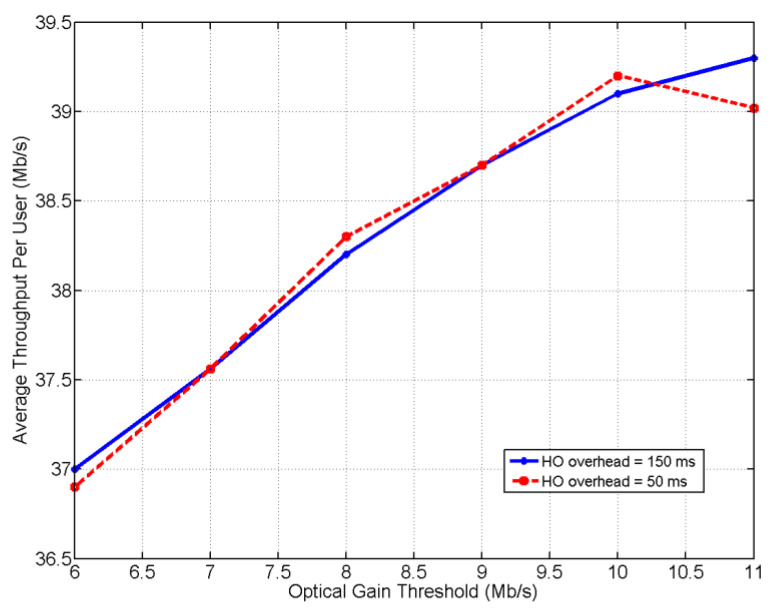
Achieved system throughput with the optical gain threshold.

As a measure of the system’s performance and efficiency, the average throughput per user is analyzed, assuming the HO overhead (HO_o_) values are 50 ms and 150 ms. To understand the HO*_p_* in Figure 11, the average throughput is evaluated with the corresponding to the γ as shown in Figure 12. With the increase of γ, higher data rates are achieved. The change in the HO_o_ does not affect the achieved throughput in the proposed system because the location of the vehicle is supposed to be fixed at the time of pairing and detection after the time of APA (lines 3, 4, and 5 in the SDA-LNV algorithm). It can be concluded from Figure 11 and Figure 12 that the user is expected to remain assigned and connected to the LiFi AP as long as the value of γ is fair at the receiver of the user, which means the user would be linked to WiFi in case of blockage or shadowing.

**Figure 13:**
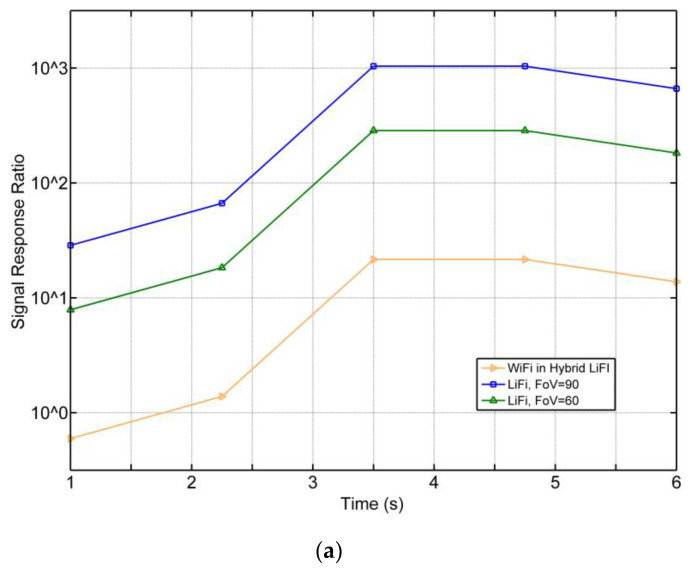

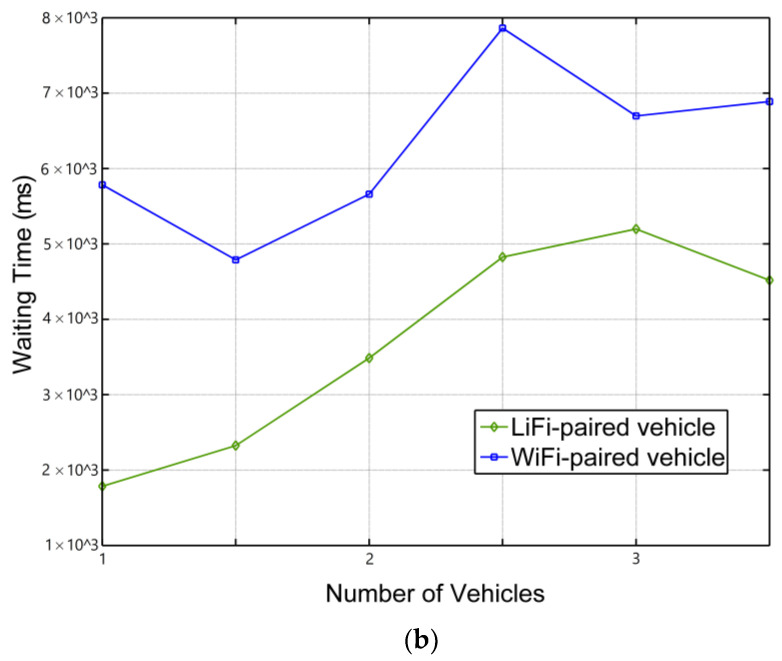
System response with LiFi and WiFi. (**a**) the signal response over time, and (**b**) vehicle waiting time (queuing time).

It is important to understand the responsiveness of the system for the used wireless technologies in the proposed system which contribute to the speed and delay between the vehicles and the CU through the infrastructure. The vehicle response to the system over time is represented by the WiFi and LiFi APs in this system. However, the responsiveness of the inner entities, such as those inside the vehicle and the CU with the smart building, is beyond the scope of this work. Figure 13a shows the signal response ratio for WiFi and LiFi in the system over time where LiFi outperforms WiFi. The responsiveness of WiFi starts lower than 10^1^, while LiFi starts around 10^2^ to 10^3^; this comes from the nature of the high speed and capacity provided by LiFi network. This enables the system to operate efficiently with reduced delay. Figure 13b shows the expected average waiting times for vehicles at the time of pairing and entrance.

**Figure 14:**
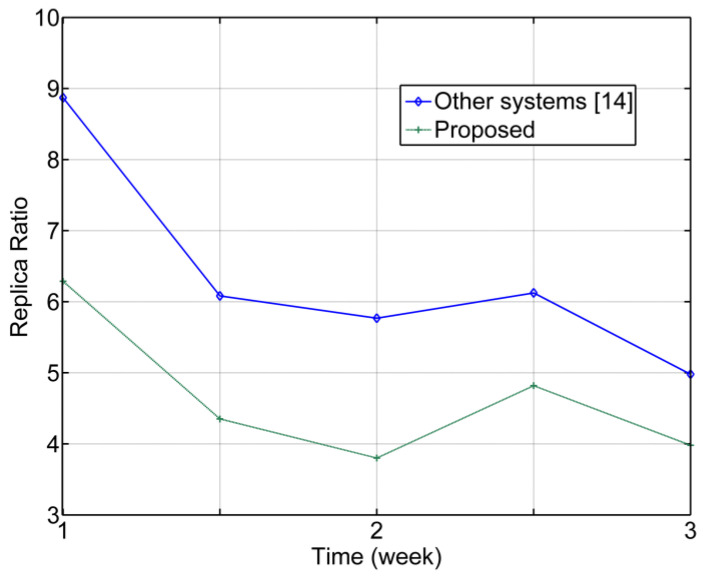
The replica ratio *Ro*.

The replica ratio Ro for COVID-19 is measured based on the expected efficiency of the proposed system. Figure 14 illustrates how using this system is expected to reduce the infection rates by weeks, where after only three weeks, the Ro is decreased drastically. The reason behind this is because when a new system is proposed to fight a newly occurring pandemic, users may take time to become familiar with using the system and following the procedure smoothly. When compared with other systems, the proposed design isolates vehicles, and reduces density and interactions among people by increasing the distance through monitoring health conditions and scheduling the authorized vehicles according to a pre-defined setting. Reducing the infection rates basically relies on the monitoring of the period of testing, where it is not possible for users to hide their health status and must follow the procedure because it is compulsory for all drivers. On the other side, in other SD systems, users may avoid some protocols or change their data. This means tricking the system is not an option for our system design. This increases the reliability of deploying it in any smart building. Different buildings may have experienced a slightly different performance of the proposed algorithm, signal response, or achieved throughput due to a variety of reasons such as the size of the building, hardware and infrastructure configurations, and training levels of the staff and users. The high efficiency of the proposed system comes from the new concept introduced for social distancing areas.

## 5. Challenges and Limitations

The proposed system design introduced in this study has several challenges, some of which are explained as follows:

I. The proposed system design is not suitable for public places because of the high cost of implementation with millions of vehicles. The privacy of people’s location would be violated in case of the system being adopted by the public sector.
II. To preserve privacy, all parties involved in this system must consent to be part of the system and be fully aware of all functionalities and specifications.
III. High cost for buildings with multiple entries and more vehicles if adopted by public sectors.
IV. For the system to be fully functional, all testing centers should be included in the database that is connected to the server.
V. In the case of using this system in public places, it would be difficult to recognize the number of individuals inside the vehicle.

## 6. Conclusions

To restrict the transmission of the lethal disease known as the new Coronavirus, it is necessary to identify infected individuals as well as those who have had intimate contact with an infected individual and then take appropriate actions. Taking this basic perspective into consideration, different SD approaches have been proposed so far, demonstrating tremendous success in lowering infection rates but with major privacy complications. Moreover, no SD technique/method exists for vehicles, especially for those who have access to buildings, and no study has employed LiFi in any SD technique so far. In this article, we have proposed a novel SD system design that avoids privacy issues and coordinates vehicle access to smart buildings. The proposed solution uses a hybrid LiFi network as a medium of transmission to provide a high-speed, secure, efficient, and reliable system that is capable of managing and reducing vehicle density inside buildings in a systematic manner.

The proposed system works with the data from COVID-19 test centers through a cloud-based CS. The system aims to monitor and detect vehicles and permit/deny access based on drivers’ status and vehicle count inside the building. The system requirements and specifications make it suitable for private companies and government sectors and not for public companies/market use. The SD approach for limiting the number of vehicles, SDA-LNV, is proposed for the first time in the social distancing area in this article with a new system design and concept. The results show the HO probability with the optical gain of the LiFi APs, and the achieved throughput (data rates) over different HO overhead values. Moreover, signal response and waiting times of vehicles were discussed and explained for a better understanding of the quality of the proposed system. Finally, the replica ratio of the infections of COVID was anticipated in this study based on the efficiency, speed, response, and careful design of each step included in the system. Using a smartphone app and web app for user–system interaction provides accurate information delivery, which enhances smart building management and keeps a healthy environment.

## Figures and Tables

**Table 1:** Benefits of using LiFi technology compared with other technologies in this work.

Metrics	LiFi	WiFi	BLE	RFID
Speed	Fast	Medium	Slow	Slow
Security	High	Low	Low	Low
Interference	Low	High	High	High
Bandwidth	Unlimited	Limited	Limited	Limited
Power consumption	Medium	High	High	Medium
Range of spectrum	High	Low	Low	Low
Privacy	Higher	Lower	Lower	Lower
Cost	Cheap	Expensive	Expensive	Expensive
Availability	Wide	Limited	Limited	Limited
Environmental impact	Lower	Higher	Higher	Higher
Health	Safe	Harmful	Harmful	Harmful

**Table 2:** Summary of related social distancing works and this work.

Ref.	Year of Publication	Scenario	Usage	Technology	Approach	Environment	Internet	Vehicle	Smartphone (Yes/No)	Phone App (Yes/No)	Smart Building
[21]	2021	RTM	Compulsory	Cloud/Fog	VSM	BCE	Needed	NO	NO	NO	NO
[22]	2021	RTM	Compulsory	IoT, Thermal and/or IR	PhD, VSM	P	Not Needed	NO	NO	NO	NO
[23]	2020	RTM, M&P	Compulsory	WiFi Video surv., IoT	MWP, CD	SC	Needed	NO	YES	YES	NO
[24]	2020	RTM	Not Compulsory	AI, Cloud/Fog, WiFi, Thermal	VSM	SHC	Needed	NO	YES	YES	NO
[25]	2020	RTM, KD	Compulsory	BLE	VSM	AC	Not Needed	NO	YES	COULD BE	NO
[26]	2021	RTM, KD	Compulsory	Cloud/Fog, IoT, WiFi, BLE	NK	IPP	Needed	NO	COULD BE	COULD BE	NO
[27]	2012	RTM	Compulsory	Unknown	MG	GB, LCL	Needed	NO	COULD BE	COULD BE	NO
[28]	2020	RTM	Compulsory	IoT, Cloud/Fog, AI, WiFi	SmH	HC	Needed	NO	COULD BE	COULD BE	NO
[29]	2021	KD	Not Compulsory	WiFi, BLE, Thermal/IR	MWP	OT	Not Needed	NO	COULD BE	COULD BE	NO
[30]	2020	KD	Compulsory	WiFi, BLE	MWP	UniC	Not Needed	NO	NO	NO	NO
[31]	2020	KD	Compulsory	UWB	PhD	IPP	Not Needed	NO	COULD BE	COULD BE	NO
[32]	2021	KD	Compulsory	ILS, WiFi, BLE	IL	IPP	Needed	NO	YES	YES	NO
[33]	2022	KD	Not Compulsory	BLE	SmH	IPP, SHC	Not Needed	Robot	COULD BE	COULD BE	NO
[34]	2022	RTM, KD	Compulsory	Video surv.	PhD	SM	Not Needed	Robot	NO	NO	NO
[35]	2022	RTM, KD	Compulsory	IoT, Video surv., GPS, cloud	MWP, TC, VSM,	OT	Needed	Drone	COULD BE	COULD BE	NO
[36]	2022	RTM, KD	Compulsory	GPS, Cloud, Thermal, IoT	TT, CD	OT, SC	Needed	Drone	YES	NO	NO
[38]	2022	InC	Compulsory	GPS	CD, TT	G	Not Needed	Drone	YES	NO	NO
[39]	2022	RTM, InC	Compulsory	Cloud, IoT, Video surv.	MWP	OT, P, G	Needed	Drone	NO	NO	NO
This work		RTM, KD, Sch	Compulsory	WiFi, LiFi, BLE, Cloud	MWP, TT, TC, SmH	LSM, OT	Needed	YES	YES	YES	YES
Definitions			Approach	MWP: monitor and warn people, VSM: vital sign monitoring, TT: travellers tracing, TC: traffic control, PhD: physical distancing, CD: crowd density, MG: mass gathering, SmH: smart health, IL: indoor localization;							
			Environment	OT: outside travelling, UniC: university campus, G: general, LSM: inside large smart buildings, P: public places, IPP: indoor public areas, SM: shopping markets, SC: smart cities, SHC: smart healthcare, AC: aircraft, GB: global, LCL: local, HC: healthcare;							
			Scenario	RTM: real-time monitoring; M&P: modelling & prediction; KD: keeping distance; InC: incentive; Sch: scheduling;							

**Table 3:** Parameter settings.

Parameters	Value	Parameters	Value
Software	MATLAB	PD extent	3 cm^2^
Database	MongoDB	Radiation angle half-intensity	60
Number of vehicles	15	Optical filter gain	1.0
Simulation area	40 m^2^	FoV semi-angle of the receiver	90
Quantity of WiFi AP	4	Refractive index	1.5
Quantity of LiFi AP	4	RF transmitter energy per AP	1 W
Movement model	RWP	RF transmitter bandwidth per AP	20 MHz
Area height	3.5	Optical to electric conversion efficiency	0.53 A/W
Optical energy per LiFi AP	9 W	Interval of each state	0.5 s
Modulation bandwidth of LiFi AP	40 MHz	Simulation time	5 min

## Data Availability

Not applicable.
